# Validity issues in the assessment of alexithymia related to the developmental stages of emotional cognition and language

**DOI:** 10.1186/1751-0759-3-12

**Published:** 2009-11-03

**Authors:** Hiroki Nishimura, Gen Komaki, Tetsuya Igarashi, Yoshiya Moriguchi, Sohei Kajiwara, Toru Akasaka

**Affiliations:** 1 Department of Psychosomatic Research, National Institute of Mental Health, National Center of Neurology and Psychiatry, Tokyo, Japan; 2 National Hospital Organization Iou National Hospital, Kanazawa, Japan; 3 Morioka Children's Hospital, Morioka, Japan

## Abstract

**Objective:**

We examined developmental aspects of the emotional awareness of adolescents by evaluating their responses to a self-reported questionnaire based on the Toronto Alexithymia Scale-20 (TAS-20).

**Methods:**

The items of the TAS-20 were modified to make them more understandable by adolescents, and nine new items related to a limited capacity for imagination were added. The Japanese Linguistic Ability Test and the multi-dimensional empathy scale for adolescents were administered to examine concurrent validity. Two hundred and two normative young adolescents and thirty-two adolescent patients with psychosomatic and/or behavioral problems participated in the study. Eighty junior high school students also participated in a separate examination of test-retest reliability.

**Results:**

Thirteen items were extracted after exploratory and confirmatory factor analyses, and four core factors were identified in the resulting scale: Difficulty Identifying Feelings (DIF), Difficulty Describing Feelings (DDF), Externally-Oriented Thinking (EOT) and Constricted Imaginal Capacities (CIC). Interestingly, scores on the multi-dimensional empathy scale correlated positively with DIF and DDF, but negatively with EOT and CIC. Higher DDF scores were associated with higher Japanese linguistic abilities. DIF/DDF scores were higher for females than males, irrespective of linguistic ability. Test-retest reliability coefficients were significant. The patient group showed significantly higher DIF scores than the normative students.

**Conclusion:**

The present findings indicated that subjective difficulties in identifying and describing feelings are associated with empathetic and linguistic abilities. The developmental aspect to emotional awareness herein described suggests that self-reported questionnaires for alexithymia must be carefully constructed and examined, even for adults.

## Introduction

The number of teenagers with symptoms of psychological stress and associated behaviors is increasing in Japan. For example, teenagers may suddenly become violent, or complain of physical symptoms (e.g., headaches and stomachaches) that do not have a clear physical cause. It has been hypothesized that such phenomena may be related to a limited ability to recognize and verbalize feelings or emotions. Indeed, aggressive children report more difficulties with understanding their emotions [1], and such difficulties are associated with somatic symptoms [2-4].

A limited ability to recognize and verbalize emotions is generally referred to as alexithymia [5], literally "no words for feelings". This concept was derived from observations of adult, psychosomatic patients, and was first introduced by Sifneos [6]. It is defined by four facets: Difficulties in identifying feelings and discriminating between emotions and physical sensations, difficulties in describing feelings, constricted imaginal capacity, and an externally-oriented cognitive style [6,7]. A widely used and extensively validated self-report questionnaire for evaluating alexithymia in adults is the twenty-item Toronto Alexithymia Scale (TAS-20). The TAS-20 has a three-factor structure [8,9]: Difficulty identifying feelings (DIF), difficulty describing feelings to others (DDF), and externally-oriented thinking (EOT).

It has been reported that alexithymia has a developmental aspect: Teenagers appear to have more difficulty identifying and describing their feelings than do adults [10] and their ability to recognize and understand emotion generally develops with age [11]. However, only a few studies to date have assessed alexithymia in children and adolescents [12,13]. These studies found that difficulties with identifying and describing feelings are associated with the somatic complaints of children [12], and that adolescents showed alexithymic characteristics to the same degree as adults (i.e., the prevalence was similar) [13]. However, these two studies have several limitations: First, neither factor loading nor internal consistency were reliable for EOT, similar to findings for adult subjects [7]. Second, the TAS-20 does not assess a limited capacity for imagination. Third, an examination of the criterion-related validity of the TAS-20 was not done. Finally, no clinical samples were examined and compared to normal subjects.

Thus, the purpose of this study was to assess alexithymia in adolescents and to compare the results to the conventional findings for adults as assessed by TAS-20. Items were added to the TAS-20 that are intended to measure limited capacity for imagination. The criterion-related validity of this new questionnaire was examined by comparing scores with those on a measure of empathic ability. Because empathy has been generally defined as "the ability to understand and share in another's emotional state or context" [14], self-awareness is necessary for its development [15]. Indeed, alexithymic individuals are relatively deficient in empathy [16-18]. As opposed to the deficit seen in people with alexithymia, there may be a strong correlation between the empathy and emotional awareness of people who are not. We thus hypothesized that there would be negative correlations between scores on our TAS-20-based alexithymia questionnaire and empathic ability. Third, we also examined the contribution of linguistic ability to the alexithymic trait, given that children's linguistic abilities are quite possibly related to their emotional understanding [1,19,20]. Finally, we examined patients with psychosomatic complaints who were of the same age as the healthy subjects to examine the discriminative validity of our questionnaire.

## Methods

### Questionnaires

#### Item collection for developing the Questionnaire to Assess Alexithymia for Adolescents (QAAA)

The Japanese version of the TAS-20 [21] was linguistically modified by junior high school teachers and clinical psychologists to make it more easily understood by adolescents. In addition, nine new items were added: Three items intended to measure externally-oriented thinking, and six items intended to measure a limited capacity for imagination. All of these items were from the Japanese translation of the Toronto Structured Interview for Alexithymia (TSIA) [22], which had previously been modified into an adolescent version. The original QAAA therefore consisted of 29 items that were intended to assess the original 4-factor alexithymia construct. Each item was scored on a five-point scale, ranging from 1 (strongly disagree) to 5 (strongly agree). Of these 29 items, 14 were negatively keyed so that the scores for these items were appropriately converted before statistical analysis.

#### The Diagnostic Literacy Test Format A for Junior High School Students [23]

This diagnostic literacy test measures the Japanese literacy ability of junior high students and has adequate reliability and validity [23]. In the current study, 'reading ability' (50 items) and 'vocabulary' (30 items) were used to assess linguistic ability.

#### The Multi-Dimensional Empathy Scale for Adolescents [24]

This 30-item self-report scale for the measurement of multidimensional empathy (early through late adolescence) was developed and validated in Japan [24]. It consists of four factors: 'Empathic concern' (an other-centered emotional response resulting from the perception of another's emotional state), 'personal distress' (immature empathy [18] and a self-centered emotional response involving fear or distress that results from witnessing another's stressful circumstances or negative emotional state), 'fantasy' (the tendency to empathize with fictional characters in books and movies) and 'cognitive empathy' (the tendency to imagine the feelings or situations of another person) [24]. This scale is based on the interpersonal reactivity index (IRI) [25], and items are scored on a five-point scale ranging from 1 (disagree) to 5 (agree).

### Participants and Procedure

#### Investigation 1

Two hundred and two students from two junior high schools (total = 202 students: age range = 12-15 years old, mean age = 13.86 ± .95 years old; 102 boys: age range = 12-15 years old, mean age = 13.85 ± .97 years old, 100 girls: age range = 12-15 years old, mean age = 13.88 ± .94 years old) participated in this study. They completed a questionnaire set consisting of the original 29-item QAAA, the diagnostic literacy test and the multi-dimensional empathy scale for adolescents in a classroom setting.

An exploratory factor analysis was performed to determine the factor structure of the original 29-item QAAA. The choice of the number of factors for extraction was based on an eigenvalue >1 criterion and construability: The criteria for factor loadings included loading more than .40 on the primary factor, and loadings of less than .40 on the other factors. As reliability coefficients, Cronbach's alpha and mean inter-item correlation coefficients (MIC) were calculated for each of the factors.

Subsequently, a confirmatory factor analysis was performed to validate the factor structure of the QAAA that was suggested by the exploratory factor analysis. Goodness-of- fit was evaluated by the following three criteria, recommended by Cole and Marsh et al [26,27]: Goodness-of-fit (GFI) > .85, adjusted goodness-of-fit (AGFI) > .80, and root-mean-square residual (RMSR) < .10. We also calculated the comparative fit index (CFI) and the root mean square error of approximation (RMSEA). The global fit indices are supported by a CFI >.90 and a RMSEA < .08.

Correlations between the diagnostic literacy test and the QAAA were calculated as Pearson coefficients. In order to examine criterion-related validity, we calculated partial correlation coefficients that controlled for diagnostic literacy test scores to assess the relationship between the multi-dimensional empathy scale for adolescents and the QAAA.

Finally, gender differences across QAAA and diagnostic literacy test scores were also examined. T-tests were used for between-group comparisons. Furthermore, for gender differences, one-way analyses of covariance (ANCOVAs) were performed with diagnostic literacy test scores entered as a covariate.

#### Investigation 2

To examine test-retest reliability, the QAAA was administered twice, two weeks apart to eighty junior high school students (42 boys, 38 girls; age range = 12-13 years old; mean age = 12.60 ± .49 years old). Test-retest intraclass correlations were calculated as Pearson's correlation coefficient; r.

#### Investigation 3

The participants here were 32 adolescent patients with psychosomatic and/or behavioral problems (15 boys, 17 girls; age range = 12-15 years old; mean age = 13.59 ± .84 years old), attending clinics at the pediatrics departments of two hospitals who answered the same test package of questionnaires used in ***Investigation 1***. They were diagnosed with orthostatic dysregulation (n = 14), school withdrawal with somatic complaints (n = 10), adjustment disorders (n = 9), hyperventilation syndrome (n = 2), migraine (n = 2), eating disorders (n = 2), bronchial asthma (n = 1), breast discomfort (n = 1), recurrent abdominal pain (n = 1), abdominal bloating (n = 1), obsessive-compulsive disorder (n = 1), other anxiety disorders (n = 1), and suspected pervasive developmental disorder (n = 1). Of these 32 patients, on admission twelve had two diagnoses (e.g. orthostatic dysregulation and school withdrawal with somatic complaints) and one patient had three diagnoses (migraine, orthostatic dysregulation, and school withdrawal with somatic complaints).

To examine discriminative validity, we compared the QAAA scores of the normative students and patients, performing one-way analyses of covariance (ANCOVAs) with diagnostic literacy test scores entered as the covariate. The data sets of ***investigations 1 ***and ***3 ***were used for this analysis.

Written informed consent to participate was obtained from all of the students, patients and their parents.

### Statistic analysis

Data analyses were performed using SPSS version 11.0 and AMOS version 4.0. The level for statistical significance was set at p < 0.05.

## Results

### Factor structure of the QAAA

Exploratory factor analysis using the principal factor method with a promax rotation was performed on the 29 original items of the QAAA. Exploratory factor analysis showed a decreasing pattern of eigenvalues (7.32, 2.44, 1.76, 1.59, 1.37, 1.23, 1.09, 1.00,.95,...) and suggested an eight-factor solution with the eigenvalue >1 criterion. However, we adopted a four-factor solution in consideration of theoretical construability because there were factors with too few items. Of the 29 items, sixteen that had factor loadings of less than .40 or cross-loadings of greater than .40 were eliminated. Consequently, thirteen items were extracted, and these 13 items were subjected to factor analysis using the same procedure. Exploratory factor analysis showed a decreasing pattern of eigenvalues (4.36, 1.71, 1.37, 1.07, .74,...). Finally, a four-factor solution was adopted by the eigenvalue >1 criterion and theoretical construability. The resulting four-factor solution accounted for 50.43% of the variance in the data. Table 1 shows the results of this exploratory factor analysis.

**Table 1:** Factor loadings, Cronbach's α Coefficients, and Inter-factor correlations of the Questionnaire to Assess Alexithymia for Adolescents (QAAA) by exploratory factor analysis

	**DIF**	**EOT**	**CIC**	**DDF**	**Communality**
*Difficulty Identifying Feelings (DIF; α = .79)*					
When I am upset, I don't know whether I am sad, frightened, or angry.	***.82***	.07	-.08	.02	.69
I am often confused about sensations in my body.	***.75***	.00	-.08	-.01	.59
I often don't know why I am angry.	***.67***	.09	-.02	.02	.42
I have physical sensations that even doctors don't understand.	***.49***	-.33	.20	.05	.43
					
*Externally-Oriented Thinking (EOT; α = .75)*					
I learn much about myself on the basis of my feelings. (R) (23)	.01	***.79***	-.05	.14	.49
It is important to be aware of one's own feeling and to understand what feeling it is. (R)	.10	***.60***	.28	.00	.54
I find examining my feelings useful in solving personal problems. (R)	-.09	***.56***	.02	-.06	.42
When I am at loss as to how to behave, I often rely on my feelings to help guide my actions. (R) (15)	.09	***.54***	.03	-.21	.42
					
*Constricted imaginal Capacities (CIC; α = .73)*					
I can become absorbed and imagine some things in the same way as I experience a good movie or story. (R) (24)	.01	-.08	***.80***	-.09	.52
I become absorbed in thinking about the characters in novels or movies. (R) (16)	.01	.05	***.67***	-.08	.63
I think I have much imagination. (R) (4)	-.12	.13	***.54***	.20	.37
					
*Difficulty Describing Feelings (DDF; α = .67)*					
It is difficult for me to reveal my innermost feelings, even to close friends.	.04	-.07	.11	***.76***	.41
It is difficult for me to find the right words for my feelings.	.02	.08	-.10	***.62***	.62

Inter-factor correlations					
DIF	---	-.35***	-.34***	.42***	
EOT		---	.42***	-.37***	
CIC			---	-.21***	

Items marked with (R) are reverse-scored. ***p < .001

The first factor (four items) accounted for 29.79% of the variance; the factor loadings ranged from .49 to .82. This factor we labeled 'Difficulty Identifying Feelings (DIF)'. The second factor (four items) accounted for 9.52% of the variance; the factor loadings ranged from .54 to .79. This factor we labeled 'Externally-Oriented Thinking (EOT)'. The third factor (three items) accounted for 6.79% of the variance; the factor loadings ranged from .54 to .80. This factor we labeled 'Constricted Imaginal Capacities (CIC)'. Finally, the fourth factor (two items) accounted for 4.34% of the variance; the factor loadings ranged from .63 to .76. This factor we labeled 'Difficulty Describing Feelings (DDF)'.

Positive correlations were found between DIF and DDF (r = .42, p < .001) and EOT and CIC (r = .42, p < .001). However, DIF was negatively correlated with EOT (r = -.35, p < .001) and CIC (r = -.34, p < .001), and DDF was negatively correlated with EOT (r = -.37, p < .001) and CIC (r = -.21, p < .001).

Cronbach's alpha were .79 for DIF, .75 for EOT, .73 for CIC, and .67 for DDF. MIC were .48 for DIF, .43 for EOT, .48 for CIC, and .50 for DDF.

A confirmatory factor analysis was conducted to validate the four-factor structure identified by the exploratory factor analysis (Table 2). The indices were .92 for GFI, .88 for AGFI, .08 for RMSR, .94 for CFI, and .07 for RMSEA, that confirmed the four-factor structure extracted by the exploratory analysis. All factor loadings were significant and of appropriate size (from .57 to .82).

**Table 2:** Factor loadings and Inter-factor correlations by confirmatory factor analysis of the Questionnaire to Assess Alexithymia for Adolescents (QAAA)

	**DIF**	**EOT**	**CIC**	**DDF**
*Difficulty Identifying Feelings*				
When I am upset, I don't know whether I am sad, frightened, or angry.	.82***			
I am often confused about sensations in my body.	.77***			
I often don't know why I am angry.	.65***			
I have physical sensations that even doctors don't understand.	.57***			
				
*Externally-Oriented Thinking*				
I learn much about myself on the basis of my feelings. (R) (23)		.59***		
It is important to be aware of one's own feeling and to understand what feeling it is. (R)		.74***		
I find examining my feelings useful in solving personal problems. (R)		.66***		
When I am at loss as to how to behave, I often rely on my feelings to help guide my actions. (R) (15)		.65***		
				
*Constricted imaginal Capacities*				
I can become absorbed and imagine some things in the same way as I experience a good movie or story. (R) (24)			.74***	
I become absorbed in thinking about the characters in novels or movies. (R) (16)			.78***	
I think I have much imagination. (R) (4)			.57***	
				
*Difficulty Describing Feelings*				
It is difficult for me to reveal my innermost feelings, even to close friends.				.66***
It is difficult for me to find the right words for my feelings.				.74***

Inter-factor correlations				
DIF	---	-.42***	-.39***	.54***
EOT		---	.57***	-.54***
CIC			---	-.37**

Items marked with (R) are reverse-scored. **p < .01, ***p < .001

### Relationships between Japanese linguistic ability and the QAAA: Gender differences

Table 3 shows correlations between diagnostic literacy test scores and QAAA scores. DDF was positively correlated with diagnostic literacy test scores, whereas EOT and CIC were negatively correlated with these scores. No significant correlations were obtained for DIF.

**Table 3:** Correlations between diagnostic literacy test scores and the Questionnaire to Assess Alexithymia for Adolescents (QAAA)

	**QAAA**			
	**DIF**	**EOT**	**CIC**	**DDF**
the diagnostic literacy test				
Reading ability	.05	-.23**	-.27***	.22**
Vocabulary	-.03	-.22**	-.26***	.26***
total score	.02	-.24**	-.29***	.25***

**p < .01 ***p < .001

Table 4 shows differences in diagnostic literacy test and QAAA scores by **sex**. Females had significantly higher total scores on the diagnostic literacy test, and also had higher DIF and DDF scores, but significantly lower EOT and CIC scores than did males. These significant gender effects on QAAA scores remained significant when diagnostic literacy test total scores were entered as a covariate (DIF: F = 9.17, df = 1, p < .01; EOT: F = 8.70, df = 1, p < .01; CIC F = 5.66, df = 1, p < .05; DDF F = 6.94, df = 1, p < .01).

**Table 4:** Comparison of gender differences in the scores of the diagnostic literacy test and the Questionnaire to Assess Alexithymia for Adolescents (QAAA)

	**male(n = 102)**		**female(n = 100)**		
			
	**mean**	**(SD)**	**mean**	**(SD)**	**t**
**the diagnostic literacy test (total score)**	**44.47**	**(14.91)**	**48.87 **	**(11.19)**	-2.37*

QAAA					
DIF	9.43	(4.08)	11.37	(3.71)	-3.47***
EOT	12.40	(3.30)	11.05	(2.78)	3.07**
CIC	8.89	(3.07)	7.57	(2.91)	3.14**
DDF	5.95	(2.21)	6.87	(1.94)	-3.13**

*p < .05 **p < .01 ***p < .001

Correlations between diagnostic literacy test total scores and QAAA scores by gender were as follows: boys; DIF, r = .04, p = n.s.; EOT, r = -.23, p < .05; CIC, r = -.43, p < .01; DDF, r = .25, p < .01, girls; DIF, r = .19, n.s.; EOT, r = -.16, p = n.s.; CIC, r = -.03, n.s.; DDF, r = .27, p < .01.

### Criterion-related validity of the QAAA

Table 5 shows partial correlations between scores on the multi-dimensional empathy scale for adolescents and QAAA scores, after controlling for diagnostic literacy test scores. The scores on the multi-dimensional empathy scale showed significant positive correlations with DIF and DDF scores, but negative correlations with EOT and CIC scores.

**Table 5:** Partial correlations of scores on the multi-dimensional empathy scale for adolescents and the Questionnaire to Assess Alexithymia for Adolescents (QAAA), after controlling for diagnostic literacy test scores

	**QAAA**			
	**DIF**	**EOT**	**CIC**	**DDF**
the multi-dimensional empathy scale for adolescents				
Emphatic concern	.24***	-.45***	-.38***	.16*
Personal distress	.40***	-.26**	-.17*	.40***
Fantasy	.40***	-.40***	-.69***	.21**
Cognitive empathy	.43***	-.55***	-.50***	.29***

*p < .05 **p < .01 ***p < .001

### Test-retest reliability of the QAAA

Test-retest reliability coefficients were as follows: r = .69 for DIF, r = .63 for EOT, r = .63 for CIC, and r = .55 for DDF (Table 6). All coefficients were significant (p < .001).

**Table 6:** Test-retest reliability coefficients of the Questionnaire to Assess Alexithymia for Adolescents (QAAA)

	**Pearson correlation coefficient s **
QAAA	
DIF	.69***
EOT	.63***
CIC	.63***
DDF	.55***

***p < .001

### Discriminative validity of the QAAA

No significant differences were found in mean age (t = .91, df = 232, p = .36) or male-female ratio (χ^2 ^= .15, df = 1, p = .70) between the normative students and patients. Table 7 shows the results of an ANCOVA in which diagnostic literacy test scores were entered as a covariate. The patient group had significantly higher DIF scores than thenormative students (F = 4.10, df = 1, p < .05).

**Table 7:** Comparison of the scores on the Questionnaire to Assess Alexithymia for Adolescents (QAAA) between normative students and patients, after controlling for diagnostic literacy test scores

	**Normative (n = 202)**		**Patients (n = 32)**		
			
	**mean**	**(SD)**	**mean**	**(SD)**	**F values**
QAAA					
DIF	10.37	(4.01)	12.09	(3.78)	4.10*
EOT	11.73	(3.12)	10.56	(2.76)	.83
CIC	8.24	(3.06)	7.53	(2.79)	.01
DDF	6.41	(2.13)	7.22	(2.12)	.96

*p < .05

## Discussion

The purpose of this study was to assess alexithymia in adolescents and to compare the findings to previous findings for adults, taking into account the contribution of linguistic ability to alexithymic traits. We also sought to examine the criterion-related and discriminative validity of the self-reported TAS-20-based questionnaire, QAAA, for adolescents.

An exploratory factor analysis found the QAAA to be comprised of four factors: DIF, EOT, CIC, and DDF. These four factors correspond to the original four facets of the alexithymia construct [6]: (a) difficulties in identifying feelings and in discriminating between emotions and physical sensations, (b) an externally oriented cognitive style, (c) constricted imaginal capacity, and (d) difficulties in describing feelings. In contrast to previous findings regarding EOT [12], we found that the factor loadings onto EOT were satisfactorily robust, thus confirming a four factor structure. This structure also has satisfactory reliability, although the MIC of DDF was slightly high, possibly because there were fewer DDF items in comparison with the other factors.

We found that DIF/DDF and EOT/CIC were negatively correlated: The higher scores should convey a deficit in cognition concerning emotions for all four factors related to alexithymia; DIF DDF, EOT and CIC. However, participants who had difficulties with identifying or describing their feelings had (paradoxically) a greater imaginal capacity and reported being more in tune with their inner experiences. These results were apparently different from the adult findings. Because positive correlations were reported between DIF/DDF and EOT among adults [8,28], this striking contrast in the current study may reflect differences in the developmental process between DIF/DDF and EOT, as we recently reported in a community sample [10]. It is possible that alexithymic adolescents, lacking skill at introspection, may overestimate their imaginal capabilities and ability to get in touch with their internal experiences. These findings, however, do not suggest that DIF and DDF scores are of no meaning; as would be the case if the items of the QAAA were not understood by the subjects, who would then answer in a more or less random way, but in fact were answered in an unexpected reverse way.

Higher Japanese linguistic ability was found to be associated with lower EOT and CIC scores but with higher DDF scores, suggesting that subjects with lower linguistic ability do not look much into their inner experiences or cultivate much imagination. In addition, positive correlations between linguistic scores and DDF scores indicated that those with higher linguistic ability attempt to describe their feelings with appropriate words. A positive correlation between DIF scores and emotional understanding [1,19] was reported among primary school-age children; however, no such association was found in the present study, suggesting that this ability has no effect on the emotional awareness of adolescents. However, it is possible that this disparity may reflect the different educational system in Japan, or perhaps difficulties with the linguistic ability questionnaire itself.

Female students scored higher for difficulties in identifying and describing feelings than did males, but showed more interest in their internal experiences and greater self-reported imaginative ability. No linguistic abilities affected these gender differences. It presents, however, doubt about whether females were not as good at identifying inner emotions, as we recently found higher DIF scores for female adult subjects [10]. These lines of evidence clearly support the validity issue of DIF/DDF factors in the self-reported questionnaire. Furthermore, regarding EOT, male adolescents were more externally-oriented, as previously reported in studies of adult males in Asian countries [10,29]. Additional cross-cultural and developmental studies on gender differences will be needed to clarify possible cultural influences on alexithymic tendencies among adolescents.

As for criterion-related validity, the various factors of the multi-dimensional empathy scale for adolescents showed negative correlations with EOT and CIC scores, consistent with previous findings with adults [18]. Students with lower levels of internal focus or with poor imaginations may have lower empathic abilities, and their attention toward psychological processes may be key in terms of the development of various aspects of emotional ability (e.g., emotional intelligence or emotional awareness) [20]. This line of evidence suggests that the cultivation of interest in inner experiences or imagination during adolescence should not be neglected.

DIF and DDF scores were positively associated with scores on the multi-dimensional empathy scale for adolescents. Those who reported having difficulty identifying and describing their feelings were more empathic, inconsistent with earlier studies of adult subjects: While high 'personal distress' scores predicted high DIF and DDF scores, high 'empathic concern' predicted low DDF scores. High 'perspective taking' (corresponding to 'cognitive empathy' in the current study) predicted low DIF scores [18]. Thus, the criterion-related validity of the DIF/DDF factors was not confirmed for early adolescent subjects using the present questionnaire, indicating that the present DIF/DDF items did not adequately assess difficulty in identifying/describing their feelings.

The items of the DIF and DDF subscales ask about one's meta-cognition: In this case, cognition about one's feelings [30]. However, meta-cognitive skill is generally still developing during adolescence [31]. Thus, as suggested by Müller et al. [32], it may be difficult to examine whether or not early adolescent subjects have identified and correctly described their feelings, especially by self-report. A high score on DIF may reflect a) actual difficulty identifying feelings that can be verified using objective measures, or b) subjective difficulty in identifying feelings that might be reported among those who have more objective talent. This view is supported by a previous report that alexithymia rates in general decreased from early to middle adolescence [13], consistent with the view that metacognitive ability develops during this time. The current unexpected results, such as the negative correlations between DIF/DDF and EOT/CIC, as well as the positive correlations between linguistic ability and DDF, may reflect inadequate metacognitive abilities among this age group. These results suggest that it is difficult to assess such characteristics in early adolescent subjects by self-reported questionnaire.

Test-retest reliability coefficients showed significant and high correlations, except for DDF. This is likely because this scale consisted of only two items. It will be necessary to develop further items for this scale for use in future studies.

The patients with psychosomatic and/or behavioral problems were significantly more likely to report difficulty in identifying feelings than the normative subjects, irrespective of linguistic ability. Consistent with previous studies, difficulty identifying feelings contributed in large part to the prediction of somatic complaints among children [12] and adults [2]. These lines of evidence do appear to support the discriminative validity of the questionnaire. However, as noted earlier, the higher DIF scores for patients do not necessarily indicate that they are alexithymic. The number of somatic complaints reported was not associated with difficulty identifying emotions, but was instead associated with the content of reported emotion (e.g., fear) [33]. Moreover, previous studies of adults and adolescents have suggested a relationship between DIF scores and depression and/or anxiety [28,34], suggesting that somatic symptoms are affected by negative mood even when alexithymia scores are high [35-37]. The TAS questionnaire appears to reflect specific aspects of depression or general distress, and a tendency to access and express negative emotions [38-40]. The evidence suggests that our depressed patients were rather self-critical, and their responses on the questionnaires may have reflected this [41].

In summary, the present findings suggest that alexithymia in early adolescents is not adequately assessed by current self-report questionnaires based on the TAS-20. This is particularly the case for the DIF and DDF subscales. In the current study more than half of the items were eliminated after exploratory factor analysis due to low factor loadings, suggesting the necessity of modifying the content of these subscales or perhaps creating a new item pool.

On the basis of our observations, we conclude that self-report questionnaires for the measurement of alexithymia in early adolescents require further careful investigation; performance measures will be needed. Moreover, the present findings also suggest that the assessment of metacognitive ability and other emotional abilities (even among adult populations) merit further research attention. Finally, although alexithymia has been considered to be a universal characteristic, the present results suggest the possibility of different developmental trajectories of alexithymia as a function of culture.

## Competing interests

The authors declare that they have no competing interests.

## Authors' contributions

HN collected and analyzed the data, performed the statistical analysis and drafted the manuscript. GK conceptualized, designed and conceived the study. TI and YM participated in the design of the study and collected the normative sample data. SK and TA collected the clinical data. All authors have read and approved the final manuscript.
